# 'Perinatal outcome in preterm premature rupture of membranes with Amniotic fluid index < 5 (AFI < 5)

**DOI:** 10.1186/1471-2393-4-15

**Published:** 2004-08-04

**Authors:** Sedigheh Borna, Hajieh Borna, Soghra khazardoost, Sedigheh Hantoushzadeh

**Affiliations:** 1 Assistant Professor of Gynecology & Obstetrics, Department of Gynecology & Obstetrics, Tehran University of Medical Sciences, Tehran, Iran; 2 Assistant Professor of pediatrics, Department of pediatrics, Shahed University of Medical Sciences, Tehran, Iran

**Keywords:** PROM, chorioamnionitis, neonatal sepsis, AFI<5

## Abstract

**Background:**

Our purpose was to determine whether AFI<5 cm after preterm premature rupture of the membranes (PPROM) is associated with an increased risk of perinatal morbidity.

**Methods:**

We performed a prospective cohort study of 95 singleton pregnancies complicated by preterm premature rupture of the membranes (PPROM) with delivery between 26 and 34 weeks gestation.

Patients were categorized in two groups on the basis of amniotic fluid index<5, (AFI<5 cm)(n = 26) or AFI ≥ 5 cm (n = 69). Categorical data were tested for significance with the χ^2 ^and Fisher exact tests. Continuous data were evaluated for normal distribution and tested for significance with the student t test.

All 2-sided p values < 0.05 were considered significant.

**Results:**

Both groups were similar with respect to selected demographics, gestational age at rupture of the membranes, gestational age at the delivery, birth weight. Both groups were similar with respect to selected variable, latency until delivery, early onset neonatal sepsis, RDS and neonatal death. Patients with AFI<5 cm demonstrated greater frequency of C/S delivery for non reassuring fetal tests (23%vs 2.8%) (p = 0.001). Our study demonstrated that patients in group I had a significant increase in the frequency of clinical chorioamnionitis (P < 0/001). Post partum infections were not seen in 2 groups.

**Conclusions:**

An AFI<5 cm after PPROM between 26 and 34 weeks gestation is associated with an increased risk of maternal infections and frequency of C/S.

## Background

Preterm premature rupture of the membranes (PPROM) is one of the most common complications of the pregnancy.

Preterm PROM is an important cause of perinatal morbidity and mortality, particularly because it is associated with brief latency from membrane rupture to deliver, perinatal infection, and umbilical cord compression due to oligohydramnios. PPROM is multifactor in nature. In any given patient, one or more path physiologic processes may be evident. Choriodecidual infection or inflammation appears to play an important role in etiology of preterm PROM, especially at early gestational ages.

It has also been proposed that amniotic fluid posse's certain bacteriostatic properties that protect against potential infections processes and that a decrease in amniotic fluid volume may impair the gravid women ability to combat such infections, this latter hypothesis was tested by Vintziuleos, et al [2].

These investigations demonstrated that patients with olighhydramnios (AFI<5), were at greater risk of having chorioamnionitis and subsequent sepsis in the neonate [2,8]. Our purpose was to determine whether patients with PPROM and amniotic fluid index <5 cm (AFI< cm) are at an increased risk of having perinatal morbidity.

## Methods

We performed a prospective cohort study of infants delivered between 26 weeks – 34 weeks gestation after preterm premature rupture of the membranes.

This study was performed at Vali-e-Asr hospital at the medical university of Tehran between October 2000 and February 2002.

All patients were at between 26 and 34 of weeks of pregnancy as best estimated by LMP, and confirmed by ultrasonography. In all patients rupture of the membrane was diagnosed by sterile speculum examination using pooled fluid, fern test and Nitrazine paper test.

Patients with clinical chorioamnionitis, nonreassuring fetal status, obstetrical indication for immediate delivery, major congenital anomalies and advanced labor (cervical dilatation >3), and a growth-restricted fetus at initial admission were excluded. 95 singleton pregnancies have observed until 34 weeks of gestation. At admission, all patients had an ultrasonographic examination, which included confirmation of the estimated gestational age, and cumulative 4 guardant AFI measurements, as previously described by Phelan et al. [3]

All patients received antibiotic prophylaxis at admission consisting of Ampicillin plus Erythromycin for 7 days following. In addition all patients received a single course of Betamethasone, consisting of two 12 mg Betamethasone injections during the first 24 hours after admission. Fetal surveillance incorporated daily non-stress testing. For fetuses with non-reassuring, non stress test results, biophysical profile assessments were performed. Indications for delivery included: labor, the diagnosis of clinical chorioamnionitis or non-reassuring fetal test results. Eligible patients were subsequently categorized into 2 groups on the basis of the admission AFI measurement. Patients in group 1 were those with an AFI<5 cm, whereas those in group 2 had AFI ≥ 5 cm. The 2 groups were compared for demographic characteristics, the estimated gestation age at both rupture of the membranes and delivery, latency until delivery, mode of delivery, birth weight, the development of clinical chorioamnionitis, postpartum endometritis, early onset neonatal sepsis and respiratory distress syndrome.

Neonatal sepsis was diagnosed by positive blood, urine, or cerebrospinal fluid cultures. Possible neonatal sepsis was diagnosed when two or more of the following criteria were present: white blood cell count less than 5000/mm3, polymorphonuclear counts less than 1800/mm3, ratio of bands to total neutrophil counts greater than 0.2. Early onset neonatal sepsis was defined as sepsis in a neonate with positive culture results or possible sepsis within the first 48 hours of life and prior to the antibiotic administration. The clinical diagnosis of chorioamnionitis was made in presence of two or more of the following criteria: maternal fever greater than 38 C, maternal tachycardia (120 beats per minute or more), leukocytosis (greater than or equal to 20,000/mm3 white blood cell), fetal tachycardia (greater than 160 beats per minute), uterine tenderness, and foul-smelling amniotic fluid.

Categorical data were tested for significance with the χ^2 ^and Fisher exact tests. Continuous data were evaluated for normal distribution and tested for significance with the student t test.

All 2-sided p values <.05 were considered significant.

## Results

A total of 95 patients with preterm premature rupture of membranes were included in the study;

26 were included in group I (AFI<5 cm) and 69 in group II (AFI ≥ 5 cm). The 2 groups were similar with respect to maternal age, Parity, and gestational age at admission (table I). Gestational age at delivery and latency period until delivery, birth weight were not significantly different between the 2 groups. Both 2 groups had similar proportions of vaginal deliveries, however, in group I cesarean delivery was more likely to be performed because of non-reassuring fetal status (table I).

**Table 1:** Demographic variable between two groups with PPPROM

Variable	AFI<5 (n = 26)	AFI ≥ 5 (n = 69)	Statistical significance
Maternal age	25,1 ± 5.2	26.3 ± 4.9	Ns
Parity	3 ± 1,5	2.4 ± 1.2	Ns
Gestational age at admission	31.5 ± 2.00	33.5 ± 1.8	Ns
Gestational age at delivery	32.6 ± 4.0	34.5 ± 3.7	Ns
Latency (Mean ± SD)	7.6 ± 4.0	6.6 ± 5.2	Ns
C/S rate for fetal distress	6(%23)	2(28%)	P = 0.001, s
Birth weight (mean)	2120 gr	2445 gr	Ns

Ns = not significant P-value < 0.05 = significant

Our study demonstrated that patients in group I had a significant increase in the frequency of clinical chorioamnionitis (P < 0/001). Post partum infections were not seen in 2 groups.

Early onset neonatal sepsis, respiratory distress syndrome (RDS), neonatal deaths were not significantly different between the 2 groups. (Table II)

**Table 2:** Maternal and neonatal outcome comparison between two groups with PPROM

Out come	AFI<5(26)	AFI ≥ 5 (69)	Statistical significant
Chorioamnionitis	5(19/2%)	2 (3%)	p < 0/001
Early onset sepsis	7(30/4%)	19(27/9%)	p = 0.819
RDS	6(26/1%)	8 (11/8%)	p = 0.1
Neonatal death	4(17/4%)	5(7/4%)	p = 0.163

P-value < 0.05 was considered significant

## Discussion

Complications of preterm premature rupture of membranes count for approximately 25% to 33% of all preterm deliveries.

Approximately, 75% of women will be delivered within 1 weeks of presentation [1,5]. More recent evidence suggests that membrane rupture is also related to biochemical processes, including disruption of collagen within the extracellular matrix of the amnion and the chorion and programmed death of cells in the fetal membranes. It has been proposed that the fetal membranes and the maternal uterine lining (decidua) respond to various stimuli, including membrane stretching and infection of the reproductive tract, by producing mediators, such as prostaglandins, cytokines, and protein hormones that govern the activities of matrix-degrading enzymes. When the fetal membranes rupture at term or before, the options are expectant management (with close observation for signs of labor, non reassuring fetal-heart-rate patterns, or intrauterine infection) or induction of labor [10,11].

Expectant management with antenatal antibiotics and corticosteroid administration are recommended the standard of care in the setting of PPROM at gestational ages of ≤ 34 [1,4,9,10]. Current evidence suggests adjunctive antibiotic therapy to reduced gestational age-dependent and infectious infant morbidity.

Amniocentesis and amniotic fluid volume have been advocated as a useful adjunct for identifying these patients [9]Several studies have implicated oligohydramnios in patients with preterm premature rupture of the membranes as a significant risk factor for perinatal infection, and fetal distress, cesarean delivery, and neonatal death [5-8,10]. In our study the finding of an AFI<5 cm after preterm premature rapture of the membranes was associated with the development of chorioamnionits. However, patients in the group with AFI<5 did not have a shorter latency until delivery. Our study did not demonstrate an association between the development of chorioamnionitis and latency interval in patients with ruptured membranes (P = 0/783), because the latency period in our study were not significantly different between 2 groups.

Other investigators have demonstrated an association between the development of chorioamnionitis and a shorter latency in patients with PPROM [5-8]. Post partum infections were not seen in our study. Perhaps, decreasing of post partum infections rates in our cases were the reason of using antibiotics after C/S. We were used intravenous Cephazolin for 48 h and then oral Cephalexin for 5 days after C/S.

Our study demonstrated that the patients with oligohydramnios were more likely to undergo cesarean delivery because of non-reassuring fetal heart rate patterns and is consistent with the findings of these other studies [5,6,8].

This study didn't show an increased frequency of early onset sepsis in the group I (AFI<5 cm), because all newborn infants in the study were treated possible sepsis with clinical symptom and laboratory evidence. 7 of the included neonates had positive blood cultures or spinal fluid cultures; as a result, there was not sensitive mechanism for appropriately determining the diagnosis of early sepsis. The negative cultures in the neonates with possible sepsis may be related to inadequate culturing techniques or the inherent difficulty encountered by most laboratories in isolating anaerobic bacteria.

Perhaps, Diagnosis of early onset neonatal sepsis and close observation for early signs of sepsis and more aggressive evaluation and early treatment for neonatal sepsis have decreased early onset sepsis in 2 groups. Preterm premature rupture of the membranes is associated with a significant decrease in the frequency of neonatal respiratory distress syndrome. In the Sims EJ study (2002), The frequency of respiratory distress syndrome in the neonate complicated with PPROM was (17%).11 This study evaluate the effect of AFI on the frequency of respiratory distress syndrome among two group that are complicated with PPROM. The frequency of respiratory distress syndrome in the neonate was not significantly lower in the group (II) than in the group I. (11/8% vs26/1%) (P < .01). The identification of oligohydramnios, defined as an AFI<5 cm, patients with preterm PPROM appear to indicate a significant risk of chorioamnionitis and early onset neonatal sepsis. These finding can aid in the counseling of patients with PPROM and may have several clinical application.

Management of PPROM requires an accurate diagnosis as well as evaluation of costs and the risks and the benefits of continued pregnancy or expeditious delivery. It is important that the patient be well informed regarding the potential for subsequent maternal, fetal, and neonatal complications regardless of the management approach.

## Pre-publication history

The pre-publication history for this paper can be accessed here:


